# A potential role for protein palmitoylation and zDHHC16 in DNA damage response

**DOI:** 10.1186/s12867-016-0065-9

**Published:** 2016-05-10

**Authors:** Na Cao, Jia-Kai Li, Yu-Qing Rao, Huijuan Liu, Ji Wu, Baojie Li, Peiquan Zhao, Li Zeng, Jing Li

**Affiliations:** Bio-X Institutes, Key Laboratory for the Genetics of Developmental and Neuropsychiatric Disorders, Ministry of Education, Shanghai Jiao Tong University, Shanghai, 200240 China; Department of Ophthalmology, Xin Hua Hospital, Shanghai Jiao Tong University School of Medicine, Shanghai, China; Neural Stem Cell Research Lab, Research Department, National Neuroscience Institute, Singapore, 308433 Singapore

**Keywords:** Protein palmitoylation, DNA damage response, zDHHC16

## Abstract

**Background:**

Cells respond to DNA damage by activating the phosphatidylinositol-3 kinase-related kinases, p53 and other pathways to promote cell cycle arrest, apoptosis, and/or DNA repair. Here we report that protein palmitoylation, a modification carried out by protein acyltransferases with zinc-finger and Asp-His-His-Cys domains (zDHHC), is required for proper DNA damage responses.

**Results:**

Inhibition of protein palmitoylation compromised DNA damage-induced activation of Atm, induction and activation of p53, cell cycle arrest at G2/M phase, and DNA damage foci assembly/disassembly in primary mouse embryonic fibroblasts. Furthermore, knockout of zDHHC16, a palmitoyltransferase gene identified as an interacting protein for c-Abl, a non-receptor tyrosine kinase involved in DNA damage response, reproduced most of the defects in DNA damage responses produced by the inhibition of protein palmitoylation.

**Conclusions:**

Our results revealed critical roles for protein palmitoylation and palmitoyltransferase zDHHC16 in early stages of DNA damage responses and in the regulation of Atm activation.

**Electronic supplementary material:**

The online version of this article (doi:10.1186/s12867-016-0065-9) contains supplementary material, which is available to authorized users.

## Background

Protein palmitoylation, or protein S-acylation, is a post-translational modification that adds a palmitate moiety to specific Cys residues by a family of proteins named protein acyltransferases (PATs) [1–4]. All PAT proteins contain a DHHC domain, a 51-amino acid Cys-rich domain with a highly conserved Asp-His-His-Cys sequence. The other regions of the PATs are variable. The zDHHC genes are numerically named zDHHC1-24 [4]. Unlike N-myristoylation or C-prenylation, S-acylation can be reversed by protein palmitoyl thioesterases and acyl protein thioesterases, making it a reversible lipid modification [5].

A number of cellular proteins have been reported to be palmitoylated, which are involved in different cellular activities such as cell signaling, protein trafficking, and cell adhesion [6–8]. Most of the palmitoylated proteins are membrane or peripheral membrane proteins, yet it is noteworthy that some non-membrane associated proteins are also palmitoylated. The importance of protein palmitoylation is manifested in zDHHC deficient mouse models. For example, mice deficient for zDHHC8 gene have increased risk of schizophrenia [9]. Mice with zDHHC13 mutation show alopecia, osteoporosis, and amyloidosis [10]. A spontaneous mutation of zDHHC21 gene in mice leads to hair loss due to defective epidermal homeostasis and hair follicle differentiation [11]. We have recently reported that mice null of zDHHC16 gene are neonatal lethal with severe heart and eye defects [12].

Recent studies have also implicated protein palmitoylation and PATs in cancer development. The expression of some zDHHC genes was found altered in various cancer tissues [13, 14]. Yet how PATs participate in cancer development remains unclear. Cancer development is driven by the accumulation of gene mutations, especially loss-of-function mutations of tumor suppressors and gain-of-function mutations of oncoproteins [15]. However, cell has a protective system, the DNA damage response (DDR), to monitor DNA damage and to repair the damage or eliminate the cells with irreparable DNA lesions [16–18]. Upon DNA damage, the cell activates the phosphatidylinositol-3 kinase-related kinases (PIKKs) such as Atm and Atr at the DNA break sites. A large number of proteins, including γH2AX and BRCT domain-containing proteins such as Brca1, TopBP1, and Mdc1 are recruited to the DNA break sites, forming transient nuclear structures named DNA damage foci, which are thought to be the centers for signal propagation and DNA repair [19–21]. Atm phosphorylates many substrates including Smad1, p53 and Chk2, which eventually cause cell cycle arrest and/or apoptosis [22–25]. Thus, a functional DDR is critical for maintaining genome integrity and preventing tumor development. On the other hand, tumor cells usually have disrupted DDR [18]. Up to date, it is not known whether protein palmitoylation plays a role in DNA damage response.

In the present study, we investigated the roles of protein palmitoylation in DNA damage response. We found defective DDR including Atm activation, p53 induction and activation, cell cycle arrest at G2/M phase, and assembly/disassembly of DNA damage foci in primary mouse embryonic fibroblasts (MEFs) in the presence of 2-bromopalmitate (2BP), a general PAT inhibitor [26–28]. These results were also observed in MEFs deficient of *zDHHC16* gene which encodes a palmitoyltransferase. These findings, for the first time, unravel an important function of PATs, in particular zDHHC16, in DNA damage response and in Atm activation, and provide a possible explanation on how zDHHC proteins participate in tumorigenesis.

## Methods

### Mice and cells

Mice were housed, bred and used in a specific pathogen free (SPF) animal facility at the Bio-X Institute, Shanghai Jiao Tong University. Specifically, no more than five adult mice were housed in one individually ventilated cage with sterilized food, water and woodchip bedding. The animal facility was maintained by professional care takers 7 days a week on a 12 h light/12 h dark cycle. The study was approved by the Institutional Animal Care and Use Committee of Shanghai Jiao Tong University [SYXK(SH)2011-0112]. Timed pregnant female mice were euthanized on embryonic E13.5 by intraperitoneal injection of over-dosed pentobarbital. The time of pregnancy was determined by visual examination of the vaginal plug in the early morning. Embryos were dissected and fibroblasts were isolated as described previously [24]. The generation and characterization of the zDHHC16 knockout mice were described in detail in our previous paper [12]. One pregnant C57Bl/6 wildtype and three zDHHC16 knockout mice were used to obtain all MEFs used in this study. The knockout mice were in mixed C57BL/6 and CBA background. All efforts were made to minimize the suffering of mice.

Cells were cultured in Dulbecco’s modified Eagle’s medium (Thermo Fisher Scientific Inc./Life Technologies, Grand island, NY, USA) containing 10 % fetal calf serum (Excell Biology Inc., Shanghai, China). They were plated at 10^6^ cells per 6 cm dish and allowed to grow overnight before any treatment. To inhibit cellular PAT activity, 2BP (2-bromopalmitate, Sigma-Aldrich, China) was used at 50 or 100 μM for 24 h as indicated. To induce DNA damage response, doxorubicin (Dox) (Selleck Chemicals, Houston, TX, USA) was used at 1 μM for different time as indicated in each experiment.

### Western blot analysis

Standard RIPA buffer containing 1 mM PMSF, 1 μg/mL aprotonin, leupeptin, and pepstatin was used for protein extraction. Protein concentration was measured using Bio-Rad DC protein assay kit (Bio-Rad Inc., Hercules, CA, USA). Western blot analysis was carried out according to the standard procedure. We used polyvinylidene fluoride membrane for protein transfer, and 5 % non-fat dried milk in PBS as the blocking agent. All primary antibodies were incubated overnight at 4 °C. Chemiluminescent detection method (ECL kit, GE Healthcare, Buckinghamshire, UK) coupled with Bio-Rad ChemiDoc XRS imaging system were used for the detection, visualization and quantitation of the proteins. All primary antibodies were purchased from cell signaling technology and used according to the provider’s instruction except for the following: anti-Atm antibody was purchased from ECM Biosciences (AM3611), anti-p-Atm antibody was purchased from Millipore (05-740) and anti-β-Actin was purchased from Santa Cruz (SC81178).

### Flow cytometry

Cells were digested with 0.25 % trypsin, washed with cold PBS and fixed in 70 % ethanol at −20 °C overnight. At the next day, cells were washed with cold PBS again and incubated in PBS containing 50 μg/mL propidium iodide (PI) and 100 μg/mL RNase A for 40 min in the dark at room temperature. The fixed and labeled cells were analyzed with Becton–Dickinson FACSCalibur (BD Bioscience, San Jose, CA, USA).

### In vitro analysis of DNA damage foci positive for γH2AX, TopBP1, and BRCA1

Cells cultured on glass slides were fixed in 4 % paraformaldehyde/PBS for 30 min followed by 0.1 % TritonX-100/PBS incubation for 40 min at room temperature. The standard immunostaining procedure was used. Specifically, 10 % goat serum was used as the blocking agent. Primary antibody incubation was carried at 4 °C overnight. Anti-p-H2AX antibody was purchased from Millipore (05-636), anti-TopBP1 antibody was purchased from BD Bioscience (611875), and anti-Brca1 antibody was purchased from Abcam (ab191042).

### Reverse transcription (RT)-polymerase chain reaction (PCR)

Trizol reagent (Invitrogen, Thermo Fisher Scientific, Grand island, NY, USA) was used to extract whole RNA and reverse transcribed using SuperScript III reverse transcriptase and random primer (Invitrogen, ThermoFisher Scientific, USA). Relative quantitative PCR was performed using the primers listed in Table 1 and the FastStart Universal SYBR Green Master from Roche (Roche Diagnostic GmbH, Mannheim, Germany). β-actin and glyceraldehyde 3-phosphate (GAPDH) were used as internal controls.

**Table 1:** The primer sequences used in relative quantitative PCR

Refseq gene	Accession number	Forward primer (5′-3′)	Reverse primer (5′-3′)	Amplicon size
Gapdh	NM_008084	TGACCTCAACTACATGGTCTACA	CTTCCCATTCTCGGCCTTG	85
Actb	NM_007393	GGCTGTATTCCCCTCCATCG	CCAGTTGGTAACAATGCCATGT	154
Zdhhc1	NM_175160	ATGAACATCTGCAACAAACCCT	GCTCCATCCATTCCTTCGAGAG	126
Zdhhc2	NM_178395	TGCTGGGCTGGTCCTACTAC	TGATAAGCCATGAGGCACAA	94
Zdhhc3	NM_178395	ATCCCCACCCATCACTTCC	CTCGGATAAACCACATGGCTC	112
Zdhhc4	NM_026917	TTACCTAGATGACGTGGGGC	AAACGATGACAAAGCCCAGT	110
Zdhhc5	NM_028379	CCGCCATATTTCTAGTGGGA	TTGCATTGTAAATGGGCACT	99
Zdhhc6	NM_144887	GAGTAAGAGGGTGGTTTCCTAGA	GCTGGATCTGAGTCACCATCAC	67
Zdhhc7	NM_025883	CACCAGGAGCCTCAGCACT	AGCATCATGGGAGCACTTGT	110
Zdhhc8	NM_133967	GGTTGGTTCCAGCACACTCT	AGAGGAAGAGGATGCCATTG	98
Zdhhc9	NM_172151	GGGCATCTTCTACCTGACCC	AGACAGCTGAACAGCCAGGT	91
zdhhc11	NM_027704	CTGACACCAATGTCCGACTCA	CGCGGTAACCTCACACAGG	116
Zdhhc12	NM_025428	GGGAATCACTCTGGTGCTCT	CCCCTTGCTCTTCCCATT	104
Zdhhc13	NM_028031	GAGGCGTGCTTGGAGAGC	CTGGAACGTGGGAGCCAT	129
Zdhhc14	NM_146073	CGGCGTCTTCTACCTGACTC	GATGGCAGGGGTGATCTTCT	100
Zdhhc15	NM_175358	TGCCAGTGCTCGTTATTGTC	AACTTTTTCCGCTGGACTCA	98
Zdhhc16	NM_023740	TACAGCTGCCAGCCTTTCC	CCCAACAGCAGACTTCGC	115
zdhhc17	NM_172554	GCGGGAGGAGGGATTTAACAC	CCCGTTTCGGTCTCGTACTC	63
zdhhc18	NM_001017968	TCAACGGGCAGACAGTGAAAC	GAAGCGGTAGTTCCGTCTCC	158
Zdhhc19	NM_199309	TGTGACACTTGTGAAGGAACC	AAAAACAGCAGCAGCGTTACA	106
Zdhhc20	NM_029492	ACCTTTGTGGTCGTCTGGTC	GCCACAAGGTAAACAACGGT	104
Zdhhc21	NM_026647	GCTGCTTACTTGCTACGCAC	CTCATGGCGAACAACAAAGA	100
Zdhhc22	NM_001080943	CGGCTGCTCAACGTGGTAG	CCAGGACGTAATTGCCCAGG	190
Zdhhc23	NM_001007460	GGCTGCCTGTTTGTGTGATTG	CCGTGATTCTTTCGCAAGTCTC	98
Zdhhc24	NM_027476	ACAGTGGCTCTCCTGCTGTT	CACACGTGTCCACCACAAA	94
Zdhhc25	NM_027306	TTGGACTTACCTCGACCCAC	ATAGGGGCAGGTAGGGACAC	109
Cdkn1a(p21)	NM_007669	TCTCAGTGTTGAATACCGTGGG	TAAGGGTAGACAGTCCAGACCA	115
Bax	NM_007527	TGAAGACAGGGGCCTTTTTG	AATTCGCCGGAGACACTCG	140
Bbc3(Puma)	NM_133234	GCCCAGCCTGTAAGATACTGT	CCTTACAGGTAGTGCCAGATGC	187

The accession number was obtained from NCBI nucleotide sequence database

### Statistical analysis

Each experiment was repeated at least three times or using cells isolated from three mutant and control mice. The results were analyzed using analysis of variance (ANOVA) with Fisher’s LSD (least significant difference) test when more than two groups were compared or unpaired t test when two groups were compared (SPSS version 18). p Value of equal or less than 0.05 was accepted as statistically significant.

## Results

### Most of the zDHHC genes are expressed in MEFs

**Fig. 1:**
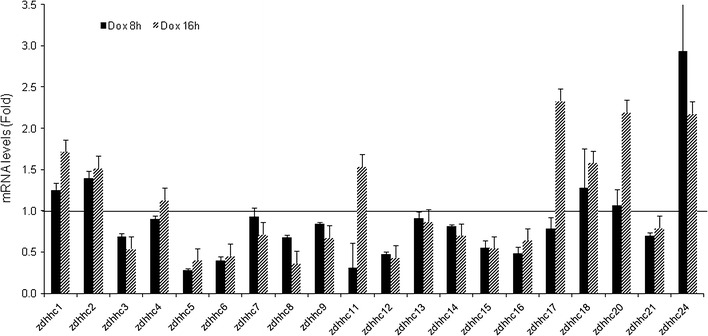
zDHHC gene expression in primary MEFs. The expression of each gene in primary MEFs without Dox treatment was set as 1 (as indicated by the *horizontal line*) and used to calculate the relative abundance of the same gene under Dox treatment for 8 and 16 h respectively. Gapdh and β-actin gene expression were used as internal controls and similar results were obtained. *Error bars* represented standard error means of repeated experiments. Notice that zDHHC10 is a pseudogene and the expression of zDHHC19, 22 and 23 genes were not detected

We chose to use primary MEFs for this study as most of the immortal cell lines show disrupted DNA damage response [18]. We first wanted to document which of the 23 protein-coding zDHHC genes were expressed in MEFs and whether their expression was altered by DNA damage. Relative quantitative PCR analysis showed that MEFs expressed 20 zDHHC genes (Fig. 1). The mRNA for zDHHC19, 22 and 23 was not detectable in MEFs. However, they were found in mouse retina tissues (data not shown). We then treated MEFs with Dox, a chemotherapeutic drug that generates double-stranded and single-stranded DNA breaks [29], and analyzed the mRNA levels of the zDHHC genes. Only zDHHC11, 17, 20 and 24 mRNA showed increase of about twofold, while the rest exhibited no significant change. The expression of a wide spectrum of PATs in primary MEFs suggests that protein palmitoylation may play important roles in these cells.

### Impaired DNA damage-induced p53 activation in the presence of 2BP

We then wanted to study the possible roles of protein palmitoylation in DNA damage response. Since it was unfeasible to simultaneously silence most of the PATs with interference RNA, we used 2BP, a substrate analog inhibitor that had been widely used to block PAT activity [30–32]. Although it may have activities other than palmitoylation inhibition [26], we have shown that 2BP at the concentrations of 50 μM and above was able to inhibit total PAT activities in cultured cells [12, 33]. Indeed, reduced protein palmitoylation was observed in MEFs in the presence of 50 μM 2BP (Additional file 1: Figure S1). Additionally, 2BP showed very modest effects on the expression of a few zDHHC genes (Additional file 1: Figure S2).

**Fig. 2:**
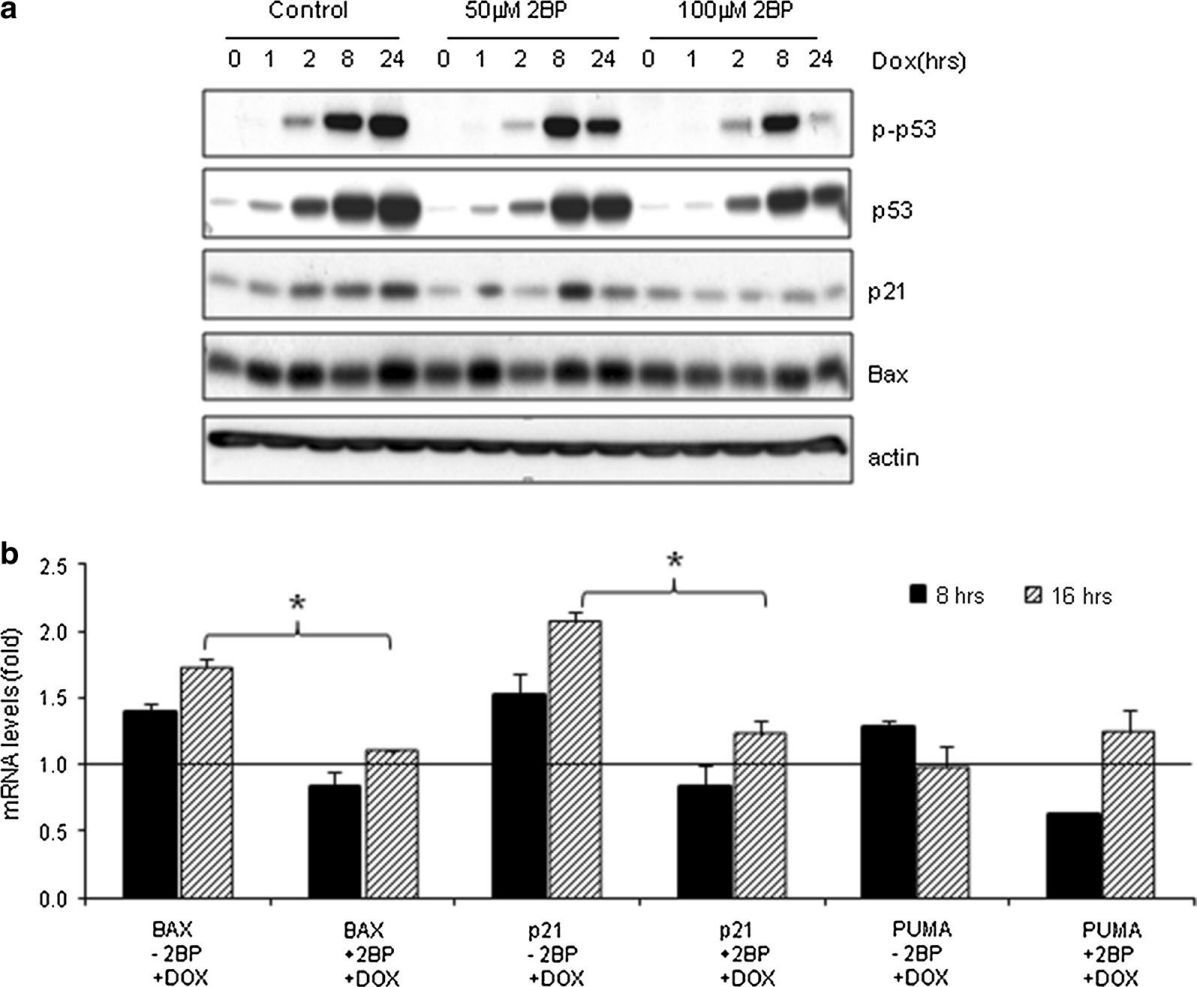
Inhibition of palmitoylation by 2BP impaired DNA damage-induced p53 up-regulation and activation. **a** Western blot analysis showed that 2BP pre-treatment impeded the induction of p53 protein expression, p53 phosphorylation on Ser15, and p21 and Bax protein levels in response to Dox treatment. Primary MEFs were pre-treated with 50 or 100 μM 2BP for 24 h and then stimulated with 1 μM of Dox for different periods of time as indicated. The protein levels of p53, p-p53, p21, Bax, and actin were determined by Western blot analysis. **b** Relative quantitative PCR results showed that 2BP pre-treatment impeded the induction of Bax and p21 (Cdkn1a) gene expression, but not much of Puma (Bbc3) gene expression. The cells were pre-treated without (−2BP) or with 50 μM 2BP (+2BP) for 24 h and followed by 1 μM Dox treatment for 0, 8 and 16 h. The expression of each gene without 2BP and Dox treatment was set as 1 (as indicated by the *horizontal line*) and used to compare the expression of the same gene under different stimulated conditions. *Error bars* represented standard error mean of the repeated experiments. *Asterisk* denoted significant difference (p < 0.05) between Dox alone and 2BP plus Dox treated samples

Western blot analysis showed that Dox-induced increase in the protein levels of p53, phosphorylation of p53 at Ser15, and the protein levels of p21 and Bax, targets of p53, were inhibited in the presence of 2BP (Fig. 2a). While Dox induced a near fivefold increase of phosphorylated p53 in normal MEFs by 24 h, the increase was only 2.5-fold or none in cells with 50 and 100 μM 2BP pre-treatment, respectively.

To further confirm these findings, we analyzed mRNA levels of p21 (Cdkn1a), Bax, and Puma (Bbc3) using relative quantitative PCR. We found that Dox treatment increased the mRNA levels of p21 and Bax but not Puma in MEFs (Fig. 2b). 2BP significantly inhibited the induction of p21 and Bax expression at the mRNA levels in response to Dox treatment (Fig. 2b). Since one of the main functions of 2BP was to inhibit PATs, these results suggest that protein palmitoylation is required for the optimal induction of p53 target genes.

### Impaired DNA damage-induced Atm activation in the presence of 2BP

**Fig. 3:**
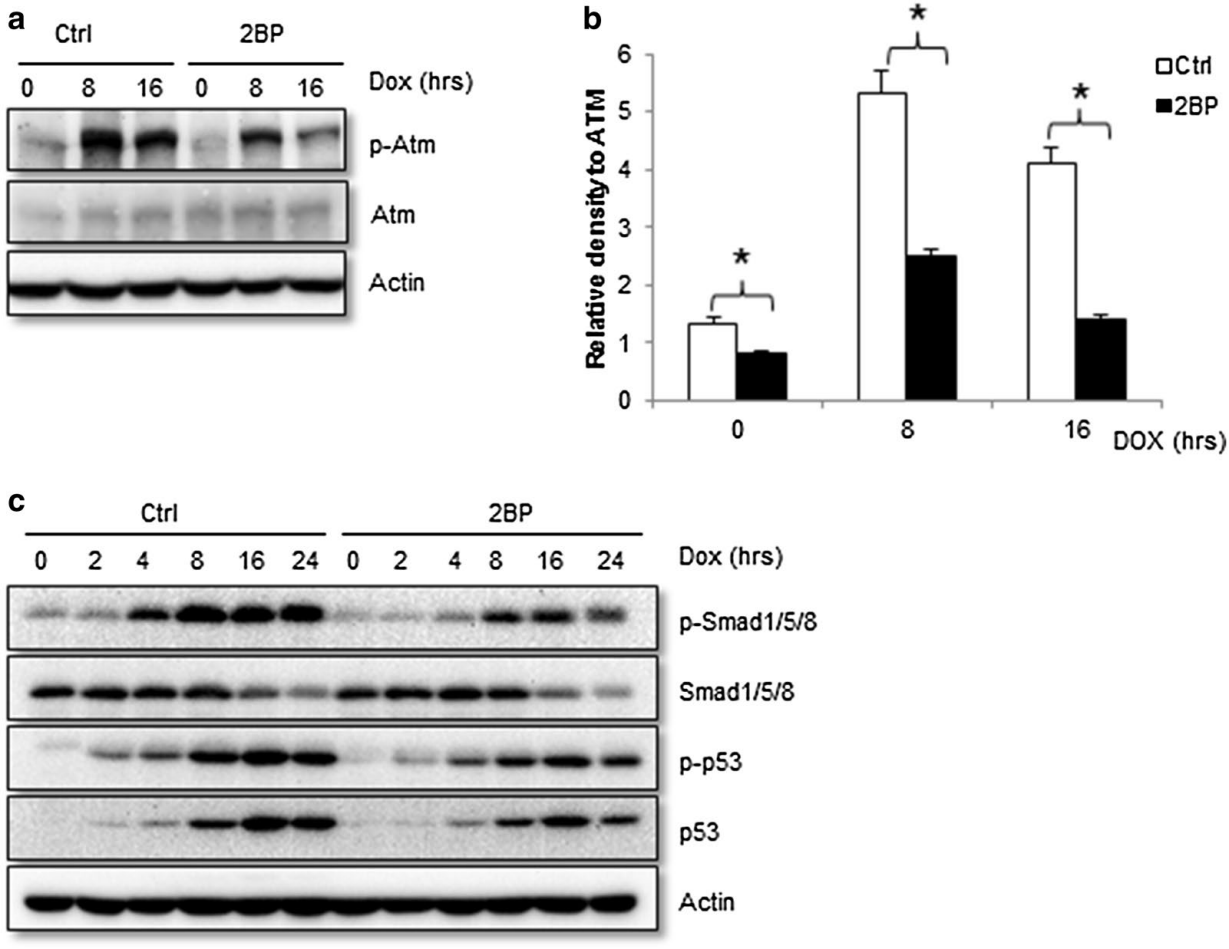
Inhibition of palmitoylation impaired DNA damage-induced Atm activation. **a** Western blot analysis showed that inhibition of palmitoyltransferase by 2BP led to a decrease in Atm phosphorylation on S1981. Primary MEFs were pre-treated with 50 μM 2BP for 24 h followed by 1 μM Dox for different periods of time. **b** Quantitation of phosphorylated Atm at Ser1981 after being normalized to total Atm. *Asterisk* denoted significant difference (p < 0.05) between control and 2BP treated cells. **c** 2BP compromised DNA damage-induced Smad1 activation. Cells were treated the same as described in 3A and probed for total and phosphorylated Smad1/5/8 and p53

The induction and activation of p53 is dependent on Atm activation in response to double-stranded DNA breaks. We found decreased activation of Atm in primary MEFs, justified by decreased Atm phosphorylation on Ser1981 (Fig. 3a, b) in the presence of 2BP. Although Ser1981 phosphorylation is not required for Atm activation in vivo, it is an autophosphorylation commonly used as an indication of Atm activation [22]. Moreover, we found that 2BP also impeded DNA damage-induced Smad1 activation (Fig. 3c), another cellular event that requires Atm activation [24].

### Impaired DNA damage foci formation in the presence of 2BP

**Fig. 4:**
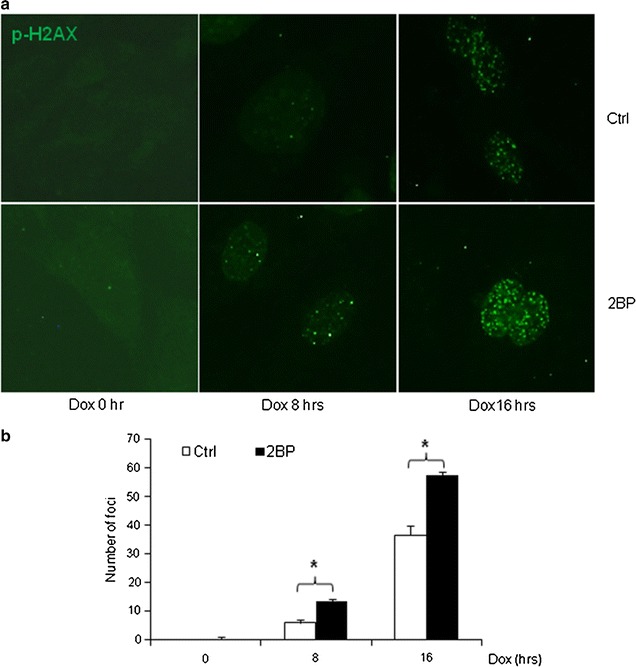
Inhibition of palmitoylation impaired assembly/disassembly of DNA damage foci. Primary MEFs grown on coverslips were pre-treated with 50 μM 2BP for 24 h and then treated with 1 μM Dox for different periods of time. The cells were then fixed and immune-stained for H2AX using Texas-red conjugated secondary antibodies (**a**). **b** Averaged number of foci per cell from multiple repeated experiments and multiple cells per experiment. *Asterisk* denoted significant difference (p < 0.05) between compared groups

We also looked at the formation of DNA damage foci in the presence of 2BP. In normal MEFs, Dox treatment induced DNA damage foci positive for γH2AX, TopBP1, and Brca1, which are believed to be signal propagation centers as well as DNA repair centers (Fig. 4a, b). In 2BP-treated cells, the number of foci positive for γH2AX was higher than those in controls, especially at 8 and 16 h after Dox treatment. These results suggest that 2BP interferes with the assembly/disassembly of the DNA damage foci or the DNA repair process.

### Impaired DNA damage-induced cell cycle arrest in the presence of 2BP

**Fig. 5:**
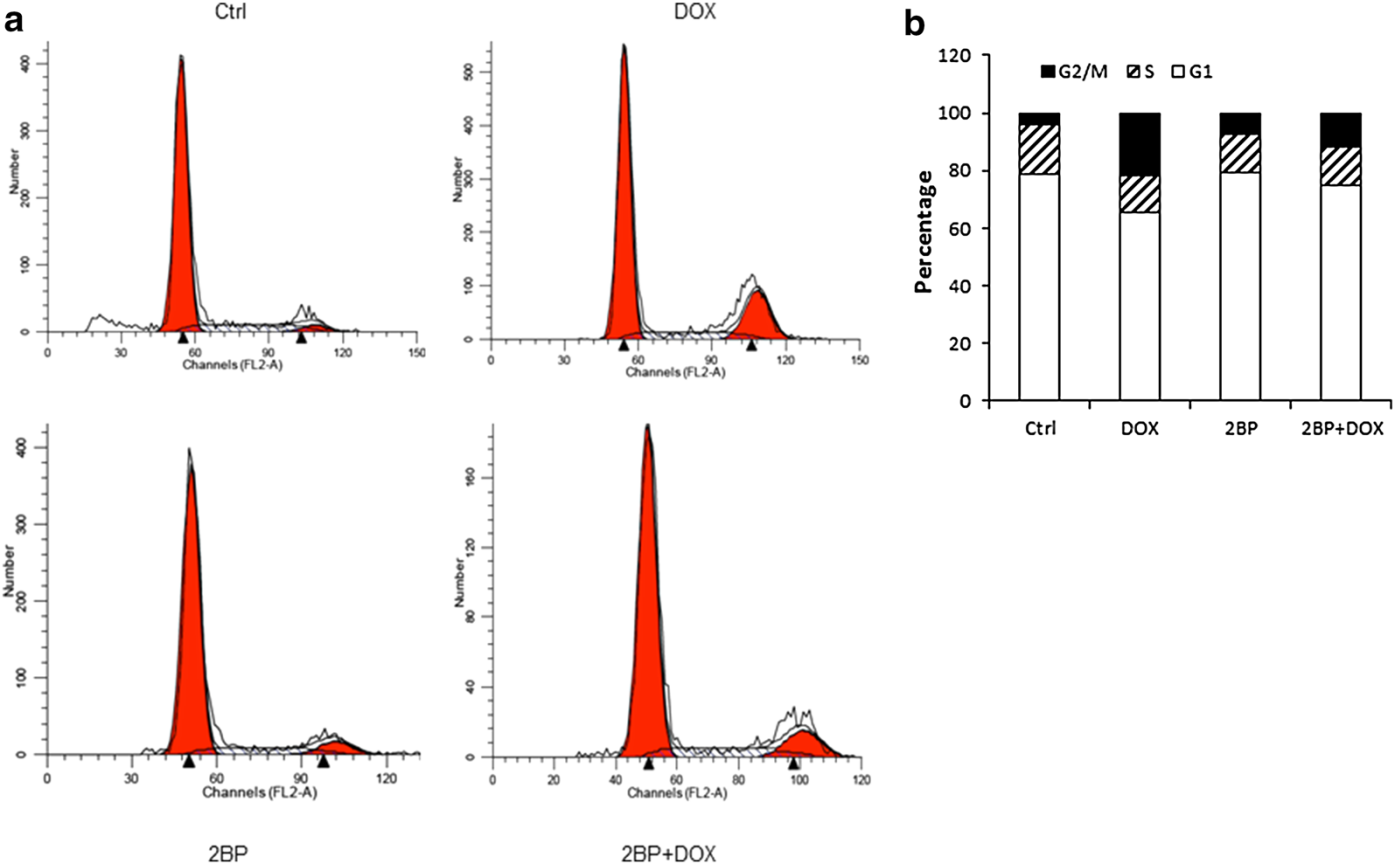
Inhibition of palmitoylation impaired DNA damage-induced cell cycle arrest at G2/M phase. Primary MEFs were pre-treated with 50 μM 2BP for 24 h and then treated with 1 μM of Dox for 24 h. The cells were harvested, fixed, stained with PI, and analyzed by flow cytometry. **a** Representative micrographs of flow cytometry analysis. **b** Averaged data from multiple experiments. The total number of cells analyzed was set as 100 % and cells at different phase were calculated accordingly

DNA damage eventually induces apoptosis or cell cycle arrest. Since 2BP compromised Atm activation and p53 induction and activation, we suspected that 2BP might affect Dox-induced cell cycle arrest and apoptosis. Cell cycle arrest was analyzed by flow cytometry (Fig. 5a). Dox treatment led to an accumulation of cells in the G2/M phase, a decrease in G1 phase cells, and a modest decrease in S phase cells in wild type MEFs. 2BP pre-treatment impeded Dox-induced increase in the percentage of cells arrested in the G2/M phase compared to control cells, without altering the percentage of S phase cells (Fig. 5b). These results suggest that protein palmitoylation is required for proper G2/M cell cycle arrest in DNA damage response. However, we found that 2BP showed some toxicity to MEFs at the doses used to inhibit protein palmitoylation, which may reflect a requirement for protein palmitoylation for cell survival, and the combination of 2BP and Dox caused more cell death (Additional file 1: Figure S3). This prevented us from further testing the role of 2BP-suppressed p53 activation in DNA damage-induced apoptosis.

### DHHC16 deficiency impaired the activation of Atm-p53

**Fig. 6:**
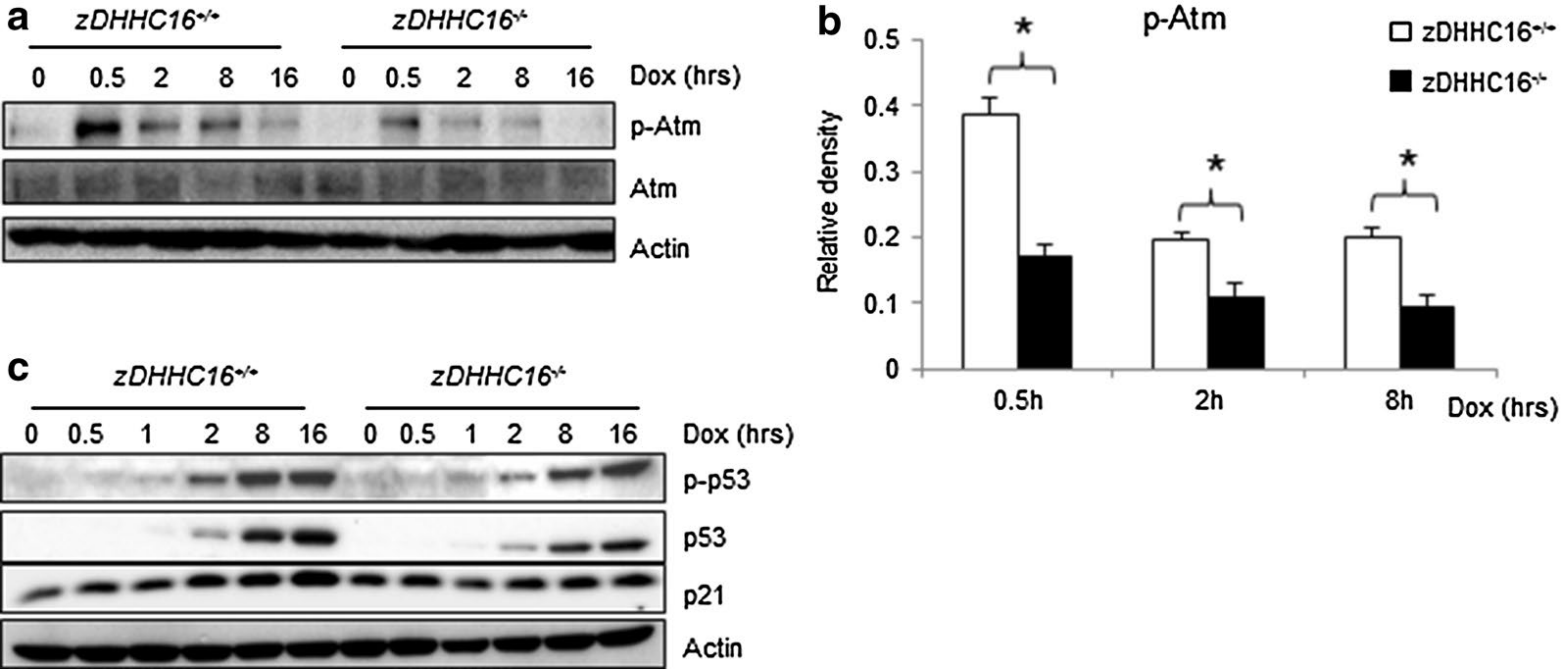
zDHHC16 deficiency impaired DNA damage-induced Atm-p53 activation. **a** Western blot analysis showed reduced Atm phosphorylation at S1981 in zDHHC16^−/−^ MEFs. Primary zDHHC16^−/−^ MEFs were isolated from zDHHC16^−/−^ mice and treated with 1 μM Dox for different periods of time as indicated. **b** Quantitation data on p-Atm. *Asterisk* denoted significant difference (p < 0.05) between control and 2BP treated cells. **c** Activation and induction of p53 and p21. Cells were treated the same as above

We have previously reported the identification of one of the PATs, encoded by zDHHC16 gene. This protein, Aph2, was originally identified as a c-Abl interacting protein (Abl-philin2) [34]. c-Abl is involved in DNA damage response. In particular, it is required for Atm-p53 activation [35–38]. Biochemical and genetic studies have shown that at least one protein, phospholamban, a cardiac muscle specific protein, was palmitoylated by Aph2 [12]. In addition, MEFs overexpress zDHHC16 also showed increased total protein palmitoylation (Additional file 1: Figure S4a). Although Dox did not affect the expression of DHHC16 at the mRNA level (Additional file 1: Figure S2), Dox induced nuclear translocation of ectopically-expressed DHHC16 (Additional file 1: Figure S4b). We have tried to raise anti-DHHC16 antibodies but those antibodies could not recognize endogenous DHHC16. This could be due to the fact that DHHC16 is a membrane protein. The lack of anti-DHHC16 antibodies prevents us to study the expression, localization, and modification of endogenous DHHC16 at the moment. To test whether DHHC16 also plays a role in DDR, we treated primary MEFs deficient for zDHHC16 gene with Dox for different periods of time. Western blot analysis revealed that zDHHC16 deficiency, like inhibition of PATs with 2BP, compromised the activation of Atm and the induction of p53 and its target protein p21 (Fig. 6a–c).

### DHHC16 deficiency impaired DNA damage foci formation and cell death

**Fig. 7:**
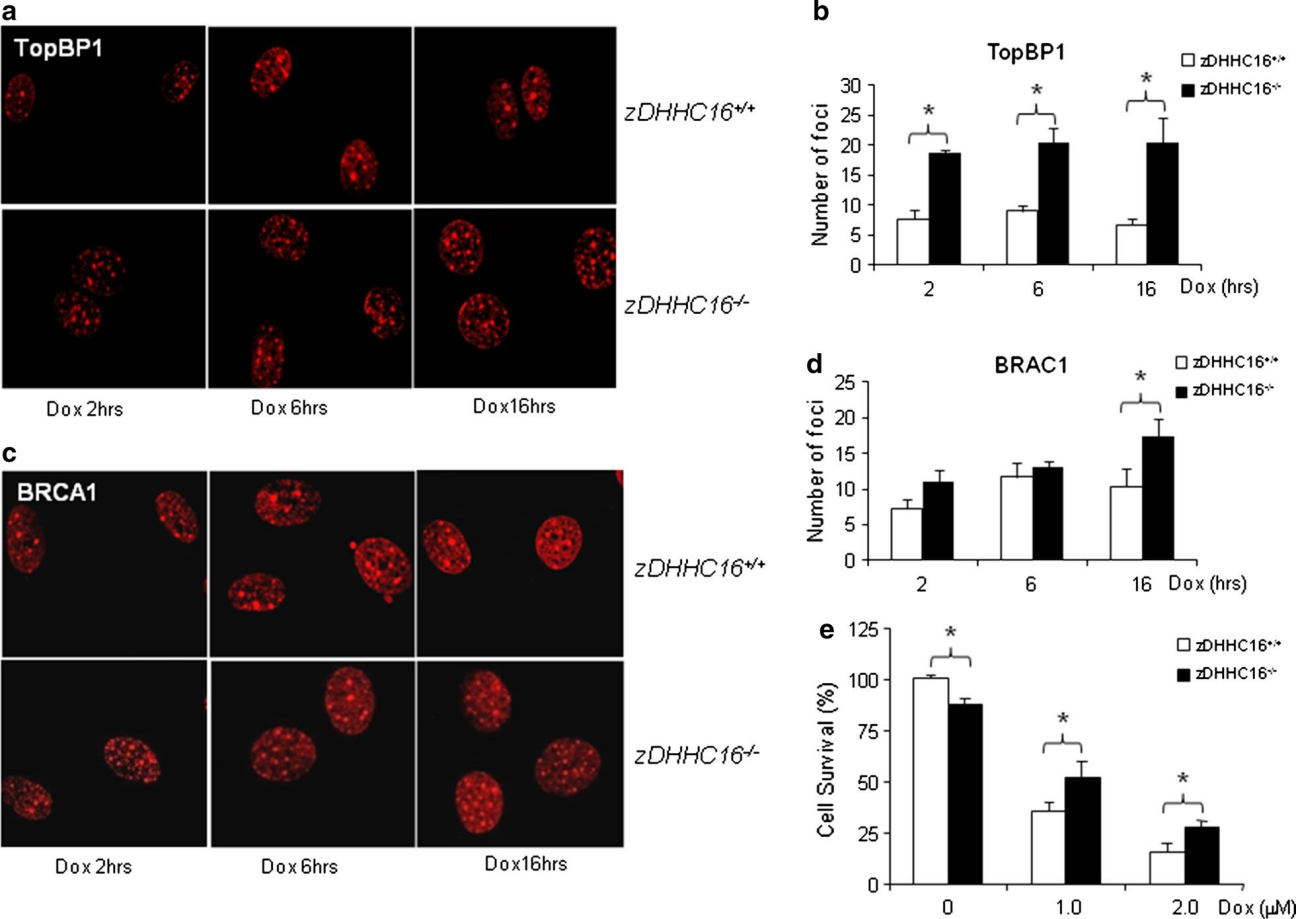
zDHHC16 deficiency impaired the assembly/disassembly of DNA damage foci. Wild type and zDHHC16^−/−^ MEFs grown on coverslips were treated with 1 μM Dox for different periods of time. The cells were then fixed and immuno-stained for TopBP1 (**a**) and BRCA1 (**c**) using Texas-red conjugated secondary antibodies. The averaged numbers of foci under each condition were counted and averaged and presented at *panels* **b** and **d**, respectively. **e** zDHHC16 deficiency resulted in an increase in cell survival in response to Dox treatment. WT and zDHHC16^−/−^ MEFs were treated with 1 or 2 μM Dox for 24 h. Cell survival rates were determined by the Wst-1 assay. *Asterisk* denoted significant difference between wild type and zDHHC16^−/−^ MEFs

We next examined DNA damage foci formation in zDHHC16 deficient MEFs. Dox-treated zDHHC16^−/−^ MEFs showed similar patterns of foci formation as the 2BP-treated wild type MEFs. The number of TopBP1 or Brca1 positive foci in zDHHC16^−/−^ MEFs was significantly higher than that in wild type cells (Fig. 7a–d). We also tested DNA damage-induced cell cycle arrest and apoptosis in zDHHC16^−/−^ and wild type MEFs. We found that Dox-induced G2/M cell cycle arrest in Aph2^−/−^ MEFs were not significantly different from those in wild type MEFs (data not shown). However, zDHHC16^−/−^ MEFs showed a modest increase in cell survival compared to wild type MEFs (Fig. 7e). These results suggest that zDHHC16 plays a role in DNA damage-induced cell death.

## Discussion

In this study, we showed that 2BP impaired Dox-induced DNA damage response, in particular the activation of the Atm-p53 pathway, and led to disrupted activation of the cell cycle checkpoint and DNA damage foci dynamics in primary MEFs. Since 2BP is a general PAT inhibitor which also binds palmitoylated proteins, we further showed that the defective DNA damage responses observed in 2BP-treated cells were largely replicated in MEFs deficient for zDHHC16, one of the 23 palmitoyltransferases. Collectively our data suggested that protein palmitoylation carried out by PATs, in particular by zDHHC16, plays an important role in DNA damage response. Since DDR, in particular the Atm-p53 pathway, is the major tumor suppression scheme, our findings also provide a possible explanation on how some zDHHC proteins exert their tumor suppression activities [13].

Palmitoylation is a common protein post-translational modification [1, 3, 14]. This is also evident by the expression of a variety of zDHHC genes in primary MEFs. However, little is known about the specific in vivo functions of each PAT. zDHHC16 knockout mice showed neonatal lethality with severe cardiac and ocular defects. It appears that one of the substrates of zDHHC16 protein in heart is phospholamban, which is largely responsible for cardiac defects observed in zDHHC16 knockout mice [12]. Here we found that cells deficient of zDHHC16 also had defective DNA damage response, a function that has not been previously ascribed to zDHHC16 or any other PATs.

How does zDHHC protein facilitate Atm activation? Since Atm is not known to be modified by palmitoylation, zDHHC16 and other PATs likely regulate the protein(s) that affect Atm activation upon DNA damage. Atm activation requires MRN complex (a protein complex consisting of Mre11, Rad50 and Nbs1), DNA conformational change, and/or DNA breaks. The generally agreed major function of palmitoylation is to increase the hydrophobicity of the targeted protein, thus facilitate protein anchoring to membrane and subsequent interaction with other proteins [1, 2, 13]. Since most of the DNA damage foci proteins and signaling molecules are localized in the nucleus, it is more likely that palmitoylation affects the stability and/or complex assembly of the proteins that are involved in DNA damage response rather than their membrane association. One such candidate may be histone protein, which has been reported to be palmitoylated [39, 40]. Palmitoylation of histone proteins may affect the remodeling of chromatin structures, which may in turn affect DNA damage foci formation and/or DNA conformation, eventually lead to alteration of Atm activation and DNA repair [21, 41]. Whether histone proteins are substrates of zDHHC16 warrants further investigation.

Alternatively, zDHHC16 protein may regulate DNA damage response through c-Abl. zDHHC16 was originally identified as a c-Abl interacting protein, which was also named Aph2 [34]. c-Abl is activated in Atm-dependent manner in response to DNA damage. Activated c-Abl helps to up-regulate p53 and p73 expression and also plays a positive role in maximal Atm/Atr activation [35–37]. zDHHC16 seems to have a similar role as c-Abl in Atm activation in DNA damage response, yet c-Abl is not a substrate of zDHHC16 [12]. It is possible that zDHHC16 may regulate DDR via c-Abl in a palmitoylation-independent manner. Further exploration on the nature of interaction between zDHHC16 and c-Abl will help understand how zDHHC16 affects DNA damage response.

## Conclusions

In summary, this study, for the first time, uncovered a critical role for protein palmitoylation and more specifically zDHHC16 in DNA damage response. These findings advance our understanding of the regulation of DNA damage response and provide a possible explanation on how protein palmitoylation is involved in cancer development. Moreover, our results expand the role of palmitoylation on cellular activities and calls for further research on palmitoylated nuclear proteins.

## Additional file

10.1186/s12867-016-0065-9 2BP inhibited protein palmitoylation in MEFs. **Figure S2.** The effect of 2BP on zDHHC gene expression in MEFs. **Figure S3.** The effect of 2BP and Dox on MEF cell survival. **Figure S4.** Overexpressed zDHHC16 increased protein palmitoylation in MEFs and was translocated into the nucleus in response to Dox.

## Abbreviations

zDHHC: zinc-finger and Asp-His-His-Cys domains
PAT: protein acyltransferase
DDR: DNA damage response
PIKKs: phosphatidylinositol-3 kinase-related kinases
MEF: mouse embryonic fibroblast
2BP: 2-bromopalmitate
DOX: doxorubicin
PI: propidium iodide
GAPDH: glyceraldehyde 3-phosphate
RT: reverse transcription
PCR: polymerase chain reaction
