# Supplementary material for: A potential role for protein palmitoylation and zDHHC16 in DNA damage response
Source: BMC Mol Biol. 2016 May 10;17:12. doi: 10.1186/s12867-016-0065-9 (PMC4862184; doi:10.1186/s12867-016-0065-9)
Supplement: Supplementary file 1 — 10.1186/s12867-016-0065-9 2BP inhibited protein palmitoylation in MEFs. Figure S2. The effect of 2BP on zDHHC gene expression in MEFs. Figure S3. The effect of 2BP and Dox on MEF cell survival. Figure S4. Overexpressed zDHHC16 increased protein palmitoylation in MEFs and was translocated into the nucleus in response to Dox. [file 12867_2016_65_MOESM1_ESM.pdf]

Additional figures S1 to S4:

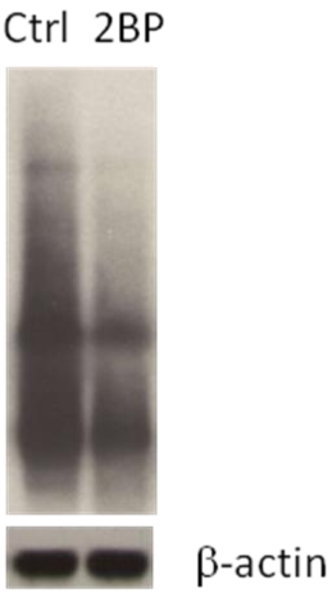

Figure S1. 2BP inhibited protein palmitoylation in MEFs.
Primary MEFs were incubated with 50 μM 2BP for 4 hours and then treated with 50 μCi [9, 10-^{3}H(N)]-palmitic acid (PerkinElmer, Waltham, MA, USA) for 24 hours. At the end of the incubation, cells were lysed and total proteins were extracted, quantitated, and resolved on 7% SDS-PAGE. The gel was fixed with acetic acid and methanol for an hour, followed by impregnating with EN^{3}HANCE™ Spray Surface Autoradiography Enhancer (PerkinElmer) for 1 hr, and wrapped and air-dried before being exposed to X-ray film for 3 weeks. A duplicate gel was transferred to polyvinylidene fluoride membraneand probed for β-actin as loading control. Ctrl: Control cells without 2BP. +2BP: treated cells with 50 μM 2BP.

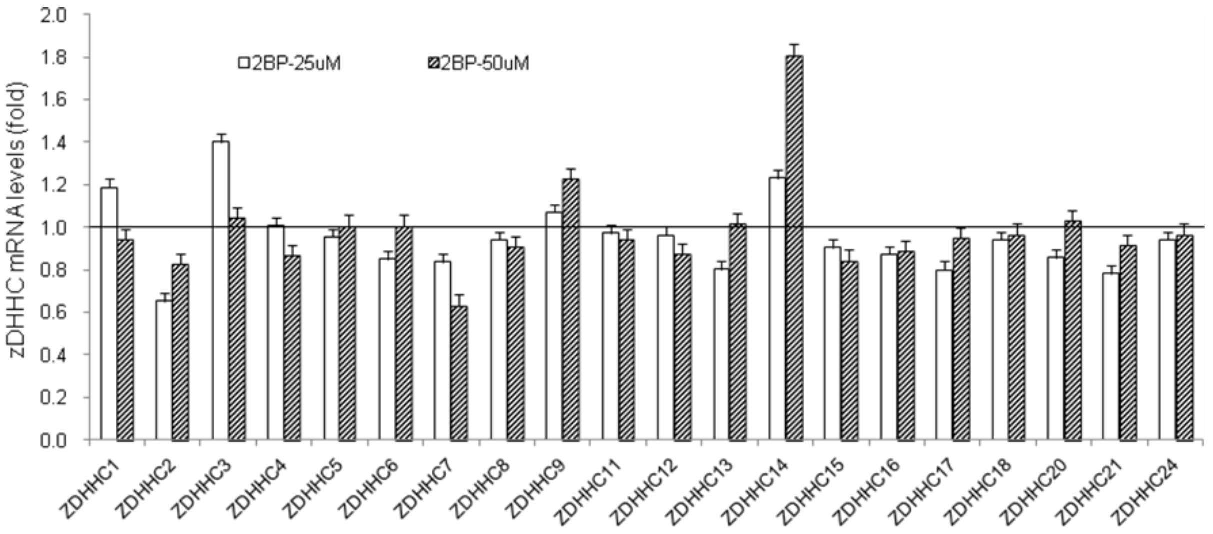

Figure S2.The effect of 2BP on zDHHC gene expression in MEFs.

Primary MEFs were incubated with 25 and 50 µM 2BP for 24 hrs. Total RNA was extracted using Trizol reagent and reverse transcribed to cDNA. The levels of zDHHC mRNA were quantitated using primers listed in Table 1. Both β-Actin and GAPDH mRNA was used as internal control and similar results were obtained. The results showed here were based on β-Actin as internal control. The expression of each mRNA without 2BP was set as 1 (as indicated by the horizontal line) and used as reference to calculate the fold change of the same mRNA under 2BP stimulations. The experiments were repeated 3 times with duplicated wells each time and the averaged results were presented. Error bars stand for standard error mean.

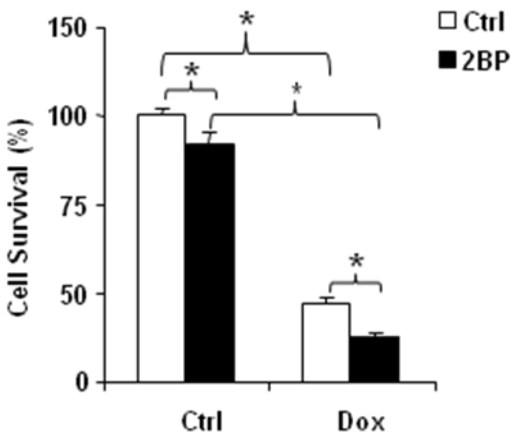

Figure S3.The effect of 2BP and Dox on MEF cell survival.

To measure cell survival rates after 2BP and Dox treatment, 10^4cells with and without 50 μM 2BP were seeded in 96-well plate and cultured overnight. They were then treated with 1 μMDox for 24 more hrs.The water-soluble tetrazolium salt(WST-1) was added to each well and incubated for 1hr at 37℃. At the end of the incubation, the plate was placed in a microplate reader and the absorbance was read at 430 nm. The absorbance of control wells without 2BP and Dox treatment was set as 100% survival and used to calculated the survival percentage of the treated cells ($OD_{treated\ cells}/OD_{controls}$*100%). The experiments were repeated three times with triplicate wells each time and the averaged survival percentage was presented with standard error means (error bars). * denotes statistically significant differences ($p<0.05$).

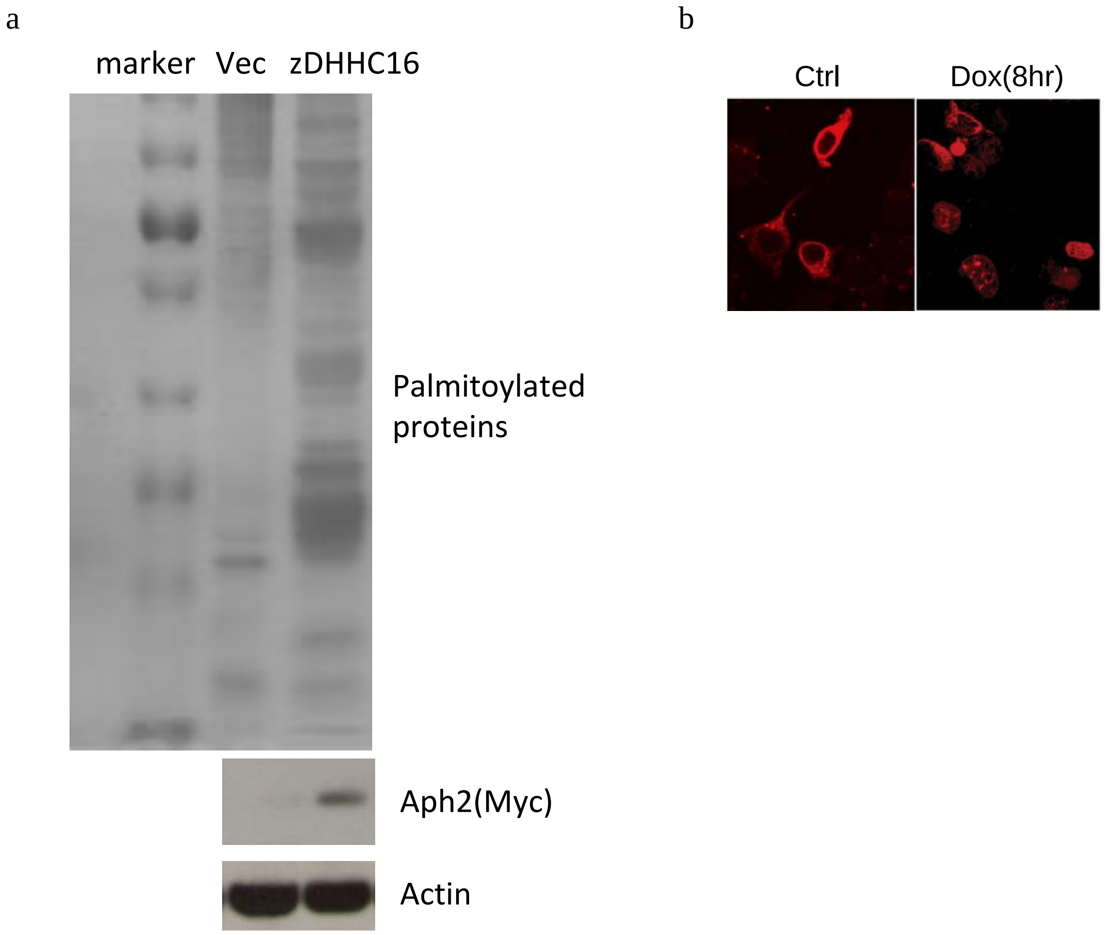

Figure S4. Overexpressed zDHHC16 increased protein palmitoylation in MEFs and was translocated into the nucleus in response to Dox.

S4a. MEFs were transfected with control vector or Myc-tagged zDHHC16 cDNA for 48 hrs. Click-it azide coupled palmitic acid (C10265, Invitrogen) was added to the culture medium for 6 hrs before the cells were harvested. About 200 μg of total protein from each experimental groups was used for the Click protein reaction (C10276, Invitrogen). The detection reagent was alkyne-coupled biotin (B10185, Invitrogen). The reaction was carried out according to the manufacturer's instruction. At the end of the reaction, palmitoylated proteins were labeled with biotin through the alkyne-azide reaction. The total protein pellet was then solubilized in SDS gel loading buffer, separated on 8% SDS PAGE, transferred to a nitrocellulose membrane and probed using streptavidin coupled with horse radish peroxidase. The labeled proteins were visualized using ECL reagents and the Bio-Rad ChemiDoc XRS imaging system. The successful transfection of the zDHHC16 cDNA was validated by a standard western blot method using anti-Myc antibody and the even loading of the gel was validated by anti-β-actin antibody. S4b. Immortalized MEFs grown on coverslips were transfected with DHHC16 (myc-tagged) expressing construct for 48 hrs and then treated with 1 μM Dox for 8 hrs. The cells were then fixed and immune-stained for Myc using Texas-red conjugated secondary antibodies. Only some cells showed DHHC16 nucleus translocation.
